# Hsa_circ_0054633 in peripheral blood can be used as a diagnostic biomarker of pre-diabetes and type 2 diabetes mellitus

**DOI:** 10.1007/s00592-016-0943-0

**Published:** 2016-11-23

**Authors:** Zhenzhou Zhao, Xuejie Li, Dongdong Jian, Peiyuan Hao, Lixin Rao, Muwei Li

**Affiliations:** 1 Department of Cardiology, People’s Hospital of Zhengzhou University, Zhengzhou University, Zhengzhou, China; 2 Department of Cardiology, The First Affiliated Hospital of Zhejiang University, Zhejiang University, Hangzhou, China

**Keywords:** Circular RNAs (circRNAs), Circulating circRNA, Type 2 diabetes mellitus (T2DM), Pre-diabetes, Microarray analysis, Biomarker

## Abstract

**Aims:**

The purpose of the current study was to investigate the characteristic expression of circular RNAs (circRNAs) in the peripheral blood of type 2 diabetes mellitus (T2DM) patients and their potential as diagnostic biomarkers for pre-diabetes and T2DM.

**Methods:**

CircRNAs in the peripheral blood from six healthy individuals and six T2DM patients were collected for microarray analysis, and an independent cohort study consisting of 20 normal cases, 20 pre-diabetes patients and 20 T2DM patients was conducted to verify the five chosen circRNAs. We then tested hsa_circ_0054633 in a third cohort (control group, *n* = 60; pre-diabetes group, *n* = 63; and T2DM group, *n* = 64) by quantitative real-time polymerase chain reaction (Q-PCR).

**Results:**

In total, 489 circRNAs were discovered to be differentially expressed between the two groups, and of these, 78 were upregulated and 411 were downregulated in the T2DM group. Five circRNAs were then selected as candidate biomarkers and further verified in a second cohort. Hsa_circ_0054633 was found to have the largest area under the curve (AUC). The diagnostic capacity of hsa_circ_0054633 was tested in a third cohort. After introducing the risk factors of T2DM, the hsa_circ_0054633 AUCs for the diagnosis of pre-diabetes and T2DM slightly increased from 0.751 (95% confidence interval [0.666–0.835], *P* < 0.001) to 0.841 ([0.773–0.910], *P* < 0.001) and from 0.793 ([0.716–0.871], *P* < 0.001) to 0.834 ([0.762–0.905], *P* < 0.001), respectively.

**Conclusions:**

Hsa_circ_0054633 presented a certain diagnostic capability for pre-diabetes and T2DM.

**Electronic supplementary material:**

The online version of this article (doi:10.1007/s00592-016-0943-0) contains supplementary material, which is available to authorized users.

## Introduction

According to the International Diabetes Federation (IDF) Diabetes Atlas (Seventh Edition, 2015), nearly 410 million diabetic patients exist worldwide, 46.5% of whom have not been diagnosed. By 2040, the number of patients with diabetes may increase to 642 million [1]. In the advanced stages of T2DM, patients often experience various complications. Therefore, early diagnosis and intervention are urgently needed. However, current diagnostic methods show various insufficiencies for the early diagnosis of T2DM. To improve this situation, researchers have assessed the value of insulin resistance, β-cell function, insulin sensitivity and fasting insulin for the diagnosis of T2DM using the homeostasis model [2]. Some researchers have attempted to identify new indicators of T2DM at early stages, and others have explored the association between genetic variants and early-onset T2DM [3, 4], a new and highly sensitive biomarker that will be of great value.

In recent years, with advancements in genomics, the single-nucleotide polymorphism (SNP) sites of related encoding sequences of some complicated diseases, including T2DM, have been gradually uncovered [5, 6]. Researchers have found that the human genome can be widely transcribed into a large number of non-coding RNAs that are closely linked to the occurrence and development of diseases [7]. CircRNAs are a type of closed circular non-coding RNAs, formed by an exon, an intron, or the reverse splicing of the two [8, 9]. Intracellular circRNAs have higher biological stability than most linear RNAs because of their resistance to RNA exonucleases [10, 11]. CircRNAs have multiple regulatory mechanisms of gene expression [12]: Some circRNAs can be used as microRNA (miRNA) sponges, playing a role in posttranscriptional regulation by engaging in competitive combination with miRNA [13]. CircRNAs can also regulate transcription by interacting with small nuclear RNA (snRNA) or RNA polymerase II in the nucleus [14] and can competitively regulate RNA splicing by binding to transcription factors [15]. Substantial amounts of circRNAs are widely distributed in the cytoplasm and nucleus [16].

CircRNAs play important roles in various diseases, including cancer, atherosclerosis, osteoarthritis, pulmonary fibrosis, myotonic dystrophy and Alzheimer’s disease [17–20]. The high biological stability of circRNAs is a precondition for their usage as biomarkers for various diseases. For example, Li et al. [21] found that hsa_circ_002059 could be used as a new biomarker for gastric cancer, Zhang et al. [22] determined that circ-ITCH could be used for the clinical diagnosis of esophageal cancer, and Qin et al. [23] discovered that hsa_circ_0005075 could be used as a potential biomarker for hepatocellular carcinoma. Regarding diabetes, cerebellar degeneration-related autoantigen 1 (CDR1) is a protein coding gene located in Xq27.1. As the natural antisense transcripts of CDR1, circRNA-CDR1 has been found to affect insulin secretion and β cell renewal [24]. In this study, we compared the expression profile of circRNAs in the peripheral blood of T2DM patients and matched control subjects by microarray analysis and then confirmed our findings in larger independent cohorts. The results demonstrated that hsa_circ_0054633 is a sensitive and specific biomarker for pre-diabetes and T2DM diagnosis.

## Materials and methods

### Study population

In this study, a total of 259 individuals were classified into three cohorts (their clinical and demographic characteristics are presented in Supplementary Tables 1–3). The participants were enrolled from among the outpatients and inpatients of the cardiology and endocrinology departments of the People’s Hospital of Zhengzhou University from July 2015 to June 2016. Subjects with any of the following characteristics were excluded: (i) malignancies, (ii) liver and kidney dysfunction, (iii) any other clinically systemic acute or chronic inflammatory disease(s), (iv) autoimmune disease, (v) untreated hypertension, and (vi) any endocrine disease other than T2DM.

### Study process

**Fig. 1:**
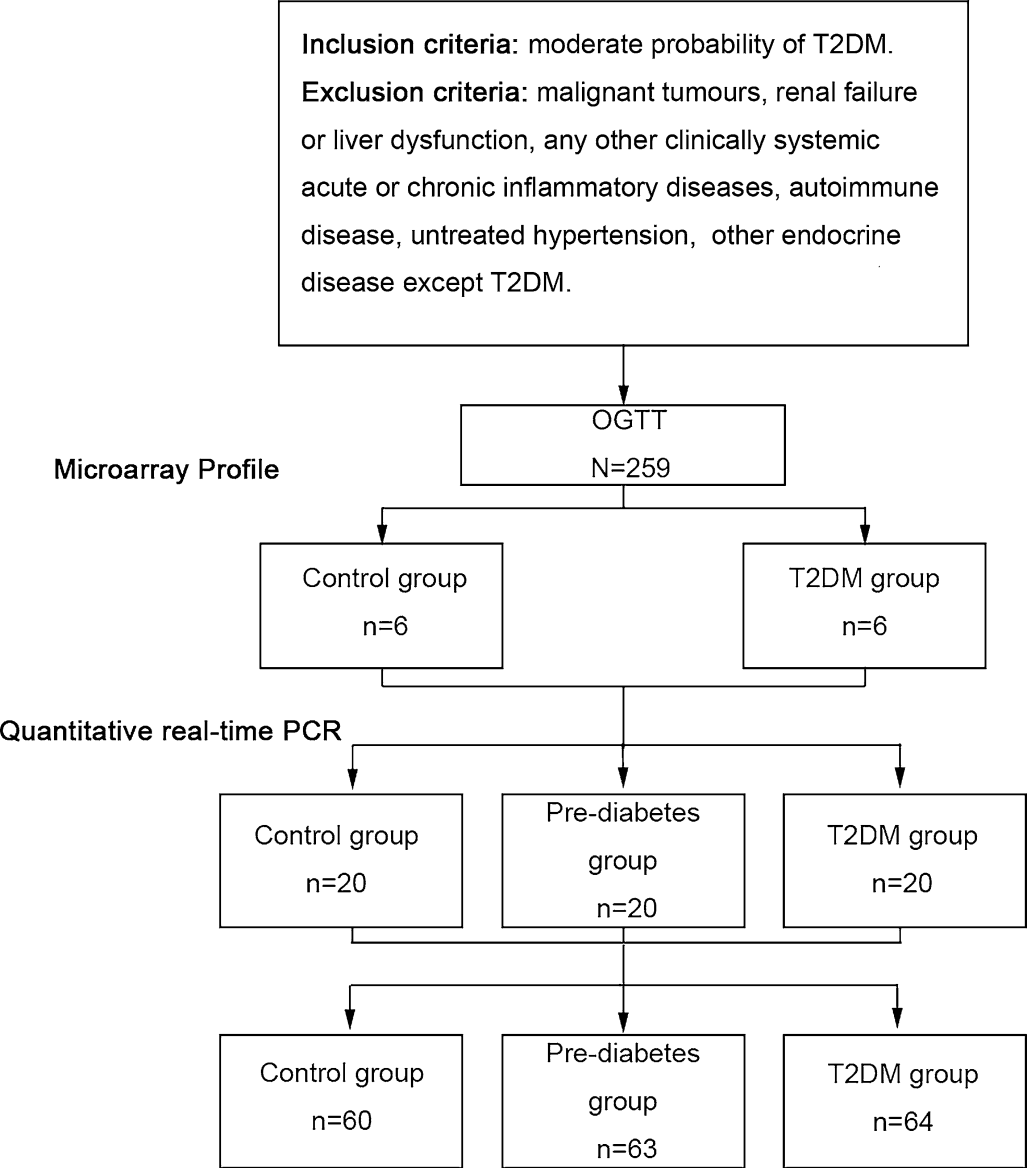
Study flow

The process of this study is shown in Fig. 1 Each study subject was tested using the oral glucose tolerance test (OGTT) to determine whether they were healthy or had pre-diabetes or T2DM. Six control individuals and six T2DM patients were selected to donate venous blood samples, and total RNA was extracted for microarray analysis. The screened circRNAs were then validated in an independent cohort (control group, *n* = 20; pre-diabetes group, *n* = 20; and T2DM group, *n* = 20). After receiver operating characteristic (ROC) curve analysis, the circRNA with the best diagnostic value was selected as the biomarker, and its diagnostic value was validated in another independent cohort (control group, *n* = 60; pre-diabetes group, *n* = 63; and T2DM group, *n* = 64).

### The definitions of pre-diabetes and T2DM and collection of whole blood samples

In this study, pre-diabetes and T2DM were diagnosed according to the 1998 Standards of the World Health Organization (WHO) [25]. Thus, patients meeting either of the following criteria could be diagnosed as having T2DM: (i) fasting plasma glucose (FPG) ≥ 125 mg/dL (7.0 mmol/L), where fasting is defined as no caloric intake for at least 8 h or (ii) two-hour post-load plasma glucose ≥200 mg/dL (11.1 mmol/L) during an OGTT.

Additionally, patients meeting either of the following standards were diagnosed as having pre-diabetes: (i) FPG ≥110 mg/dL (6.1 mmol/L) and <125 mg/dL (7.0 mmol/L); or (ii) two-hour post-load plasma glucose ≥140 mg/dL (7.8 mmol/L) and <200 mg/dL (11.1 mmol/L) during an OGTT.

Blood sample collection was performed as follows: After overnight fasting, 2 mL of blood was collected from the median cubital vein of each patient before breakfast and then stored in ethylenediaminetetraacetic acid (EDTA) anticoagulant vacutainers. The total RNA was then extracted as soon as possible.

### RNA extraction and Q-PCR

A fast total RNA extraction kit (Biotech, Beijing, China) was used to extract total RNA from 1 mL of whole blood according to the manufacturer’s instructions. RNA was then dissolved in RNase-free water. The yield and purity were measured by a NanoDrop 2000 instrument (Thermo Scientific, Waltham, MA, USA). The integrity of the RNA was determined by 1% formaldehyde denaturing gel electrophoresis. A PrimeScript RT Reagent Kit (Takara Bio, Nojihigashi, Kusatsu, Japan) was used for the production of complementary DNA (cDNA) by reverse transcription, according to the manufacturer’s instructions. Q-PCR was performed using SYBR-Green Premix Ex Taq (Takara Bio, Nojihigashi, Kusatsu, Japan) and monitored by an ABI PRISM 7500 Sequence Detection System (Applied Biosystems, Life Technologies, Waltham, MA, USA). The relative expression levels of circRNAs were determined via Q-PCR. The sequences of the primers used in the Q-PCR assay are shown in Supplementary Table 4.

### CircRNA microarray analysis

The RNAs of the peripheral blood of six control subjects and six T2DM patients were extracted for microarray analysis. The purity and concentration of the RNA were determined by a NanoDrop ND-1000 instrument (Thermo Scientific, Waltham, MA, USA). The integrity of the RNA was evaluated using a Bioanalyzer 2100 (Agilent Technologies, Santa Clara, CA, USA). The extracted RNAs were digested, dephosphorylated, denatured, amplified and labeled with Cy3-dCTP according to the manufacturer’s specifications. The purified RNAs were hybridized to a microarray (Agilent human circRNA Array V2.0) containing 170,340 human circRNA probes. The microarray data of the circRNAs were then analyzed using GeneSpring software V13.0 (Agilent Technologies, Santa Clara, CA, USA). The thresholds were as follows: fold change, ≥2 or ≤−2; *P* < 0.05 according to the *t* test.

### Data analysis

Variables with different distributions were expressed as means ± standard deviations, medians (quartiles) or percentages when they fit. In the scatterplot of circRNA expression, the horizontal lines represent the median values. The Chi-square test was used for categorical variables, whereas the Kolmogorov–Smirnov and Shapiro–Wilk tests were performed to check data normality for continuous variables, followed by the test for homogeneity of variances. The clinical and demographic indicators were checked for significant differences by one-way analysis of variance (ANOVA), if the continuous variables were consistent with the normal distribution and homogeneity of variance; if not, the Kruskal–Wallis *H* test was used. The clinical diagnostic value of a given circRNA was verified by ROC curve analysis, and when the AUC was equal to 0.5, the circRNA was defined as having no diagnostic value. Furthermore, logistic regression analysis was performed to obtain an odds ratio (OR) when the relative expression of circRNAs was expanded by ten times. *P* < 0.05 was considered statistically significant. All statistical analyses were conducted using SPSS 22.0 (SPSS Inc., Chicago, IL, USA).

## Results

### Expression profiles of circRNAs in the peripheral blood of diabetic patients

**Fig. 2:**
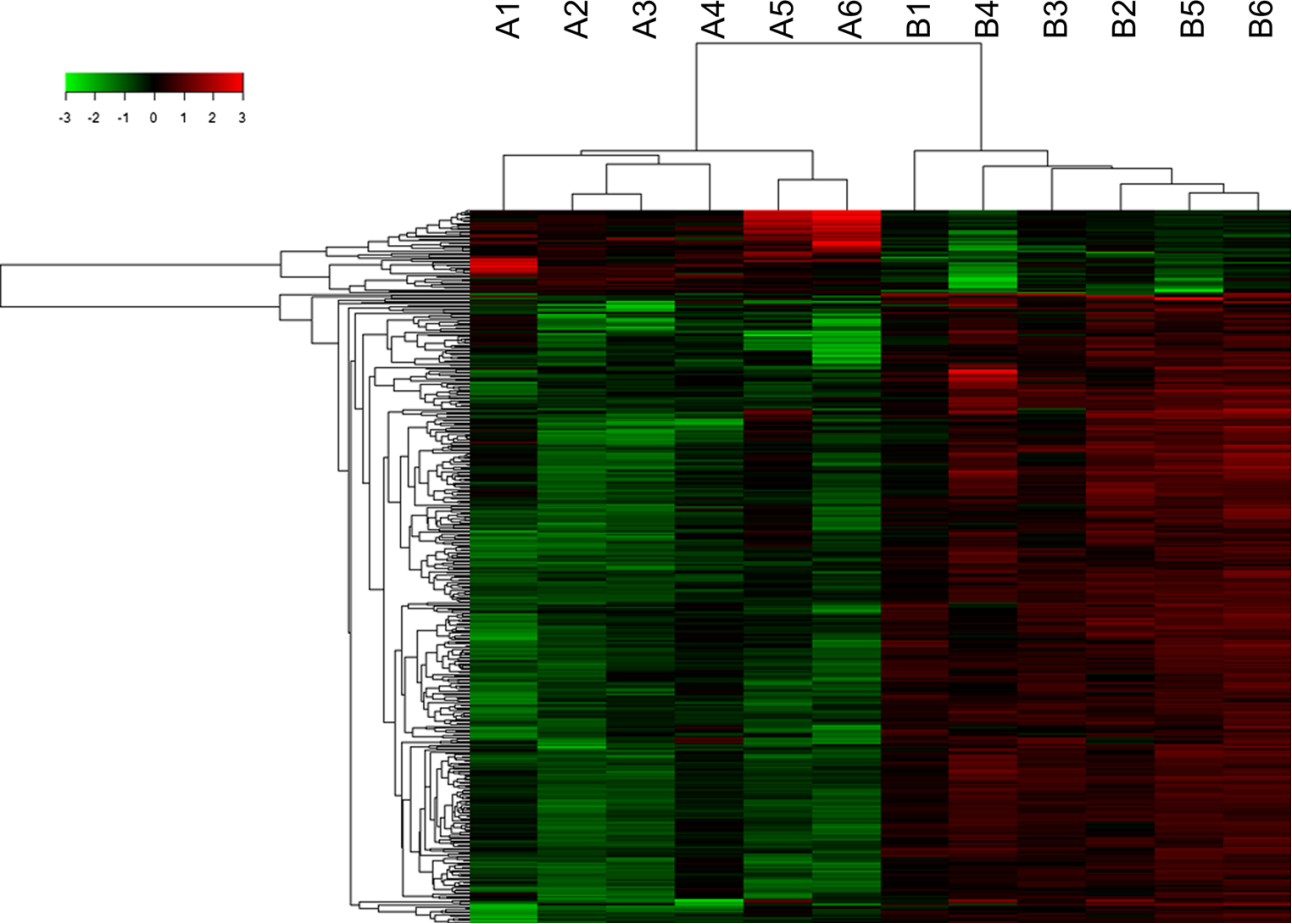
Heat map of the circRNA microarray profiles in control individuals and T2DM patients. The expression of circRNAs is hierarchically clustered on the *y*-axis, and blood samples are hierarchically clustered on the *x*-axis. The expression levels are presented in *red* and *green*, which indicate upregulated and downregulated circRNAs, respectively. *Numbers* marked with A and B are from control individuals and T2DM patients, respectively

To investigate the expression profiles of circRNAs in healthy individuals and T2DM patients, six healthy subjects and six T2DM patients were selected. Microarray analysis of the expression profiles of circRNAs in peripheral blood was performed using the Agilent human circRNA Array (V2.0). The results showed clear differences in the expression profiles of circRNAs between the two groups (Fig. 2). Differential expression was detected in a total of 489 circRNAs; of these, 78 were upregulated and 411 were downregulated in the T2DM group (Supplementary Table 5). To obtain the biomarkers that would be most applicable in clinical practice, the candidate biomarkers were selected from the 78 upregulated circRNAs utilizing stricter screening criteria: fold change >2.4 and *P* < 0.01. Five circRNAs met these standards: hsa_circ_0068087, hsa_circ_0054633, hsa_circ_0124636, hsa_circ_0139110 and hsa_circ_0018508 (highlighted in Supplementary Table 5). These circRNAs were used as candidate biomarkers in a subsequent validation utilizing a larger cohort.

### The expression profile of circRNAs verified by Q-PCR

**Fig. 3:**
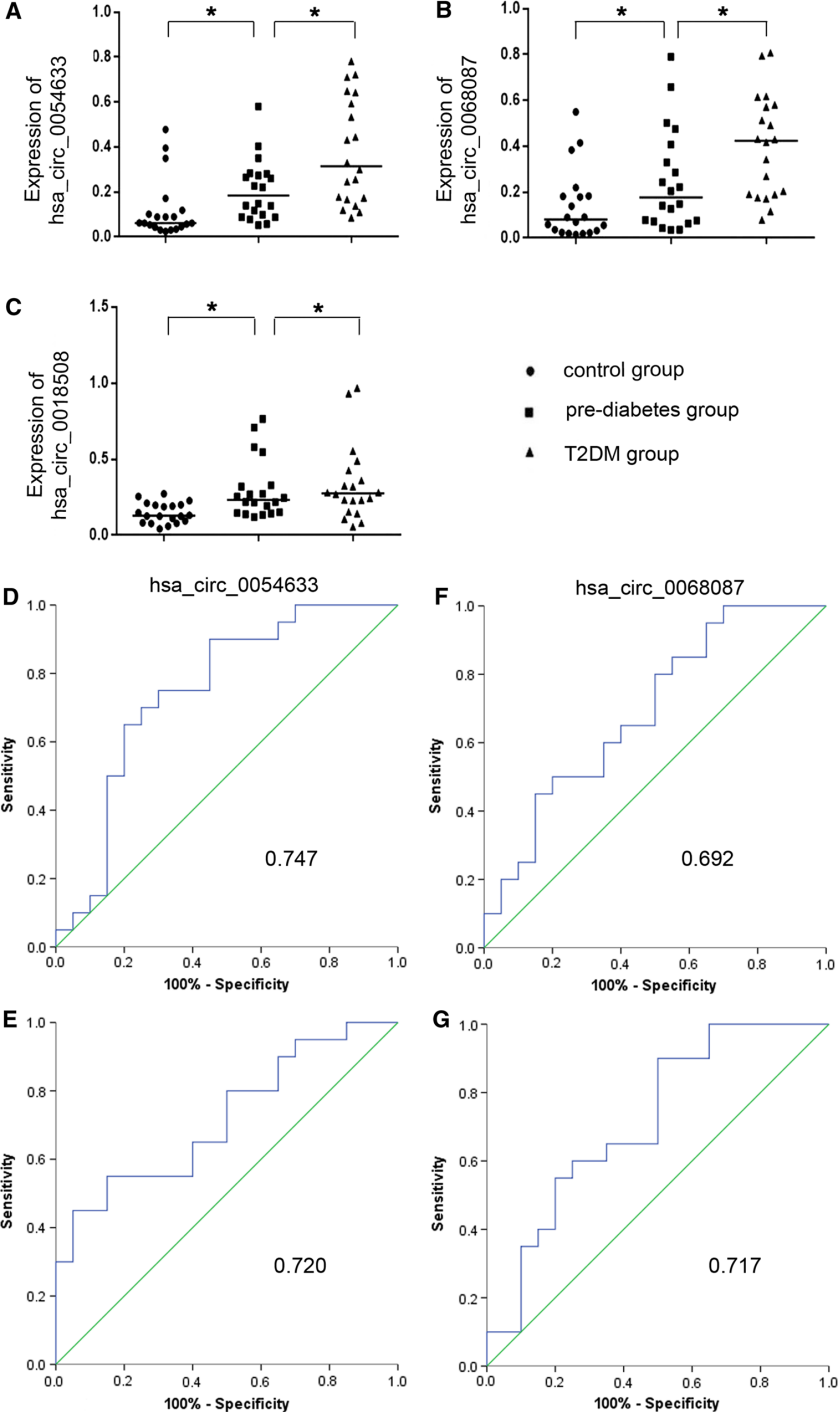
Expression levels of the selected circRNAs quantified by Q-PCR. **a**, **b** and **c** show the relative expression levels of hsa_circ_0054633, hsa_circ_0068087 and hsa_circ_0018508. *P < 0.05; **d**, **f** present the ROC curve analyses of hsa_circ_0054633 and hsa_circ_0068087 for the diagnosis of pre-diabetes; **e**, **g** are the ROC curve analyses of hsa_circ_0054633 and hsa_circ_0068087 for the diagnosis of T2DM. The AUC values are given on the graphs

To validate the five selected candidate circRNAs, Q-PCR was conducted in an independent cohort (control group, *n* = 20; pre-diabetes group, *n* = 20; and T2DM group, *n* = 20). The results are shown in Fig. 3. The levels of hsa_circ_0124636 and hsa_circ_0139110 expression among the three groups presented no significant differences. The levels of hsa_circ_0018508 expression in the pre-diabetes and T2DM groups did not differ, but both were higher than that of the control group. The expression levels of hsa_circ_0054633 and hsa_circ_0068087 were significantly different among the three groups and increased gradually from the control group to the pre-diabetes group to the T2DM group.

### ROC curve analysis of circRNAs with differential expression

To determine the diagnostic values of hsa_circ_0054633 and hsa_circ_0068087 for pre-diabetes and T2DM, ROC curve analysis was performed (Fig. 3). The AUCs of hsa_circ_0054633 for the diagnosis of pre-diabetes and T2DM were 0.747 ([0.589–0.906], *P* = 0.007) and 0.72 ([0.562–0.878], *P* = 0.017), respectively. The AUCs of hsa_circ_0068087 for the diagnosis of pre-diabetes and T2DM were 0.692 ([0.529–0.856], *P* = 0.037) and 0.717 ([0.557–0.878], *P* = 0.019); the sensitivity and specificity are shown in Table 1. Because hsa_circ_0054633 showed higher AUC and lower *P* values than hsa_circ_0068087, the former was chosen as the diagnostic biomarker for pre-diabetes and T2DM.

**Table 1:** Validation of the selected circRNAs by Q-PCR

CircRNA	AUC	95% CI	*P* value	Sensitivity	Specificity	Fold change
hsa_circ_0054633						
Pre-diabetes	0.747	0.589–0.906	0.007	0.75	0.70	1.7
T2DM	0.720	0.562–0.878	0.017	0.55	0.85	1.8
hsa_circ_0068087						
Pre-diabetes	0.692	0.529–0.856	0.037	0.80	0.50	1.8
T2DM	0.717	0.557–0.878	0.019	0.90	0.50	1.6

### Further clinical validation of the biomarker

**Fig. 4:**
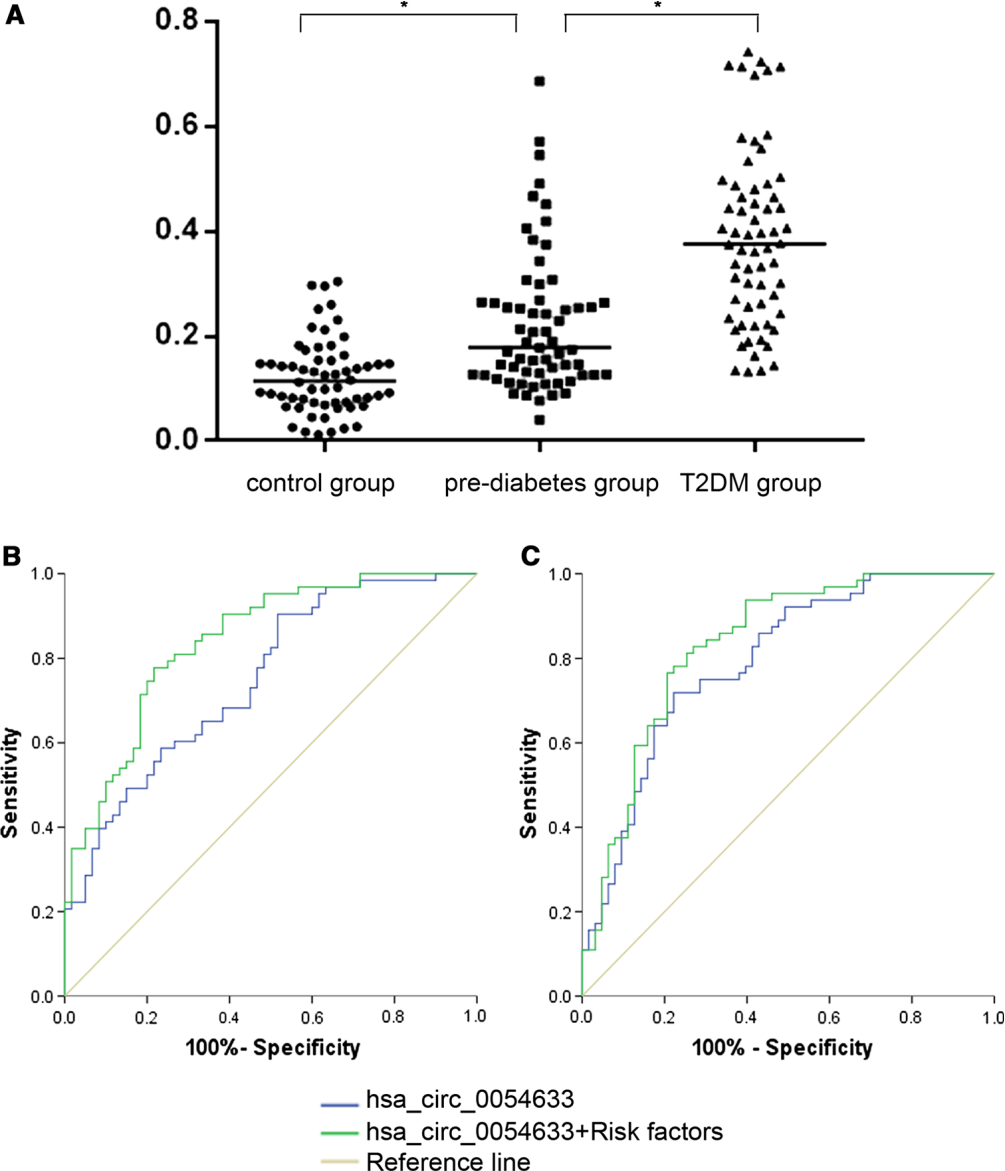
Expression levels of hsa_circ_0054633. **a** The expression levels of hsa_circ_0054633 in the control, pre-diabetes and T2DM groups. *P < 0.05. **b**, **c** present the ROC curve analyses of hsa_circ_0054633 and hsa_circ_0054633 + risk factors for the diagnosis of pre-diabetes and T2DM, respectively

To verify its clinical diagnostic capability, hsa_circ_0054633 was tested in another cohort (control group, *n* = 60; pre-diabetes group, *n* = 63; and T2DM group, *n* = 64). The results are shown in Fig. 4. The level of hsa_circ_0054633 expression increased gradually from the control group to the pre-diabetes group to the T2DM group, with a fold change of 1.8 between the first two groups and 1.7 between the latter two groups. Then, the ROC curve analysis was performed. When used as a biomarker for the diagnosis of pre-diabetes and T2DM, the AUC, threshold, sensitivity and specificity of hsa_circ_0054633 were 0.751 ([0.666–0.835], *P* < 0.001), 0.103, 0.905 and 0.483, respectively, and 0.793 ([0.716–0.871], *P* < 0.001), 0.270, 0.719 and 0.778, respectively. The crude ORs were 3.05 ([1.803–5.159], *P* < 0.001) and 2.056 ([1.530–2.762], *P* < 0.001), respectively. After introducing the risk factors of T2DM (smoking, hypertension, body mass index [BMI], total cholesterol [TC], triglycerides [TG], high-density lipoprotein [HDL] and low-density lipoprotein [LDL]), the AUCs increased to 0.841 ([0.773–0.910], *P* < 0.001) with a sensitivity of 0.778 and a specificity of 0.783 and 0.834 ([0.762–0.905], *P* < 0.001) with a sensitivity of 0.766 and a specificity of 0.794, respectively. The adjusted ORs were 6.797 ([3.025–15.273], *P* < 0.001) and 2.769 ([1.881–4.077], *P* < 0.001), respectively.

### Biomarker expression levels in different gender and age groups

To investigate the levels of hsa_circ_0054633 expression in different gender and age groups, the three groups of the third cohort were divided according to age (cut-off: 50 years old) and gender. The results are shown in Table 2 and indicate that no differential expression of hsa_circ_0054633 was identified between the different gender and age groups.

**Table 2:** Expression levels of hsa_circ_0054633 among different gender and age groups

Variable	Number	Control group			Pre-diabetes group			T2DM group	
		Expression quantity Median (quartile)	*P* value	Number	Expression quantity Median (quartile)	*P* value	Number	Expression quantity Median (quartile)	*P* value
Age (years)									
<50	37	0.116 (0.072,0.148)	0.665	34	0.199 (0.128,0.277)	0.363	32	0.414 (0.235,0.496)	0.301
≥50	23	0.102 (0.073,0.199)		29	0.155 (0.120,0.259)		32	0.350 (0.246,0.450)	
Gender									
Male	29	0.099 (0.066,0.169)	0.762	32	0.183 (0.126,0.261)	0.45	35	0.393 (0.256,0.558)	0.438
Female	31	0.133 (0.074,0.154)		31	0.170 (0.129,0.375)		29	0.364 (0.232,0.483)	

## Discussion

The high morbidity of T2DM and its various complications severely threaten human health. In the advanced stages of T2DM, patients often experience various complications, which severely impact their quality of life. Multiple large-scale investigations have revealed that intensive glucose-lowing therapy in the early phases of T2DM can benefit patients substantially, reducing the incidences of macrovascular and microvascular complications [26, 27]. However, in the early stages of T2DM, most patients are asymptomatic, and they rarely visit hospitals to seek diagnosis and therapy.

Current diagnostic methods have various deficiencies for the early diagnosis of T2DM: OGTT is the gold standard for diagnosing T2DM. However, because this procedure is time-consuming and complicated, it is only considered when there is a strong suspicion that a patient has T2DM. FPG is convenient, but the rate of missed diagnoses is high [28]. Finally, hemoglobin A1c (HbA1c) test has not been standardized in Chinese hospitals. Therefore, a convenient, highly specific and sensitive diagnostic method is urgently needed to facilitate the early diagnosis of T2DM.

Because of their convenience of sampling and low cost, hematological markers play an important role in the diagnosis of many diseases. One of the most important functions of circRNAs is their role as “miRNA sponges,” which competitively bind miRNAs to generate post-transcriptional regulation. Long non-coding RNAs (lncRNAs) can also interact with miRNAs to regulate gene translation, suggesting a potential correlation among the three types of non-coding RNAs [29]. Some lncRNAs and miRNAs have been demonstrated to be involved in the occurrence and development of T2DM; furthermore, they can be used as biomarkers for T2DM diagnosis [30, 31]. CircRNAs are much more stable than linear RNAs in cells, and in some tissues, their expression levels are ten times higher [10]; thus, circRNAs make better biomarkers.

The current study revealed significant differences between the expression levels of circRNAs in the peripheral blood of T2DM patients and that of healthy subjects. To reduce the interference of factors other than T2DM to the minimum, we strictly matched the demographic and clinical characteristics (especially the hematological indicators, such as the blood cell count and percentage of white blood cells) of the first cohort. But individual differences among the patients were huge; the differential expression of circRNA might be attributed to changes in the activation states of different blood cell types. Therefore, we selected the five circRNAs with the most significant differences as candidate biomarkers. Hsa_circ_0054633 showed the highest diagnostic value for pre-diabetes and T2DM among the five candidate biomarkers and was further verified in another cohort. It continued to have reliable diagnostic value, suggesting that hsa_circ_0054633 has the potential to be used as a diagnostic biomarker for pre-diabetes and T2DM in clinical practice.

The field of circRNAs is quite new, and thus, to the best of our knowledge, no definite evidence demonstrating the functions of hsa_circ_0054633 is available. The results of gene ontology (GO) analysis revealed that hsa_circ_0054633 not only participates in biological processes, such as cell cycle and mitotic cell cycle arrest, but is strongly correlated with molecular catabolism. The cell cycle is the basic process of cellular life activities. The proliferation of β cells is regulated by cell cycle progress, and decreased β cell proliferation is the major cause of insufficient insulin secretion [32], which is the basic characteristic of T2DM. Besides, T2DM is a chronic metabolic disease characterized by disordered carbohydrate, lipid and protein metabolism, and we hypothesize that hsa_circ_0054633 may participate in the pathogenesis of T2DM by influencing the cellular metabolism and cell cycle.

To the best of our knowledge, this study is the first to investigate the expression profiles of circRNAs in the peripheral blood of patients with T2DM and to validate the utility of hsa_circ_0054633 as a diagnostic biomarker for pre-diabetes and T2DM. The biomarker identified in this study (hsa_circ_0054633) can be easily tested using peripheral blood. Furthermore, its relatively low cost and high specificity and sensitivity make it a potentially highly useful tool for the diagnosis of T2DM and pre-diabetes.

In the present study, only hsa_circ_0054633 was validated; thus, the expression profiles of other circRNAs in T2DM and pre-diabetes patients remain to be explored. Additionally, this was a single-center study, with a high geographic concentration of subjects. Therefore, whether populations in other regions exhibit similar circRNAs expression profiles is unknown. Thus, the results of our study require further verification in larger and more diverse cohorts.

## Electronic supplementary material

Below is the link to the electronic supplementary material.

Supplementary material 1 (PDF 181 kb)

Supplementary material 2 (PDF 7 kb)

Supplementary material 3 (PDF 127 kb)
