# Supplementary material for: Hsa_circ_0054633 in peripheral blood can be used as a diagnostic biomarker of pre-diabetes and type 2 diabetes mellitus
Source: Acta Diabetol. 2016 Nov 23;54(3):237–45. doi: 10.1007/s00592-016-0943-0 (PMC5329094; doi:10.1007/s00592-016-0943-0)
Supplement: Supplementary file 1 — Supplementary material 1 (PDF 181 kb) [file 592_2016_943_MOESM1_ESM.pdf]

Supplementary Table 1. The clinical and demographic characteristics of the first cohort

	Control group (n=6)	T2DM group （n=6）	*P* value
Male gender	3（50%）	3（50%）	1
Age(years)	60±2.3	62.3±6.2	0.42
Hypertension	1（16.7%）	2（33.3%）	1
Smoker	1（16.7%）	1（16.7%）	1
BMI(kg/m^2)	23.3±2.1	24.7±1.2	0.178
TC(mmol/L)	3.5±0.9	3.9±1.1	0.506
TG(mmol/L)	0.9±0.2	1.4±0.6	0.073
HDL(mmol/L)	1.3±0.3	1.1±0.2	0.202
LDL(mmol/L)	1.8±0.7	2.1±0.9	0.559
ALT(U/L)	36.3±17.8	33.7±17.9	0.801
AST(U/L)	23.5±3.8	19.8±7.3	0.299
Scr(μmol/L)	58.7±3.9	60.0±10.7	0.781
FT4I(pmol/L)	14.4±2.7	14.1±1.9	0.801
HbA1c (%)	5.4±0.1	6.9±0.7	0.003
HbA1c (mmol/mol)	35.0±1.9	52.2±7.8	0.002
GLU(mmol/L)	5.0±0.4	9.8±2.0	<0.001
OGTT 2h(mmol/L)	7.0±0.4	13.8±1.3	<0.001

Supplementary Table 2. The clinical and demographic characteristics of the second cohort

	Control group (n=20)	Pre-diabetes group (n=20)	T2DM group (n=20)	*P* value
Male gender	8(40.0%)	11(55.0%)	12(60.0%)	0.42
Age(years)	49.6±4.7	47.2±5.9	51.1±5.5	0.083
Hypertension	5(25.0%)	6 (30.0%)	8 (40.0%)	0.583
Smoker	3(15.0%)	5(25.0%)	5(25.0%)	0.675
BMI(kg/m^2)	23.6±2.1	22.8±2.2	23.7±2.0	0.343

TC(mmol/L)	3.7±0.8	3.9±1.0	4.2±0.9	0.196
TG(mmol/L)	1.5±0.4	1.6±0.5	1.7±0.7	0.342
HDL(mmol/L)	1.3±0.5	1.1±0.2	1.1±0.3	0.501
LDL(mmol/L)	2.5±0.6	2.8±0.6	2.8±0.6	0.083
ALT(U/L)	24.8±6.6	24.1±2.9	27.7±7.6	0.415
AST(U/L)	24.9±5.6	26.1±4.4	23.2±4.5	0.169
Scr(μmol/L)	59.2±6.5	58.3±8.0	61.2±4.9	0.387
FT4I(pmol/L)	14.3±1.6	15.2±2.4	13.9±1.9	0.111
HbA1c (%)	5.0±0.2	6.0±0.2	7.5±0.7	＜0.001
HbA1c(mmol/mol)	31.2±2.6	42.1±2.2	58.2±7.1	＜0.001
GLU(mmol/L)	5.0±0.4	6.5±0.2	7.9±0.6	＜0.001
OGTT 2h(mmol/L)	6.8±0.5	8.9±1.4	12.9±1.0	＜0.001

Supplementary Table 3. The clinical and demographic characteristics of the third cohort

	Control group (n=60)	Pre-diabetes group (n=63)	T2DM group (n=64)	***P*** **value**
Male gender	29(48.3%)	32(50.8%)	35(54.7%)	0.774
Age(years)	49.0±5.5	49.0±6.6	50.6±5.7	0.222
Hypertension	21(35.0%)	25(39.7%)	31（48.4%）	0.302
Smoker	12(20.0%)	13(20.6%)	18(28.1%)	0.484
BMI(kg/m^2)	24.5±3.8	25.0±3.1	23.9±3.8	0.192
TC(mmol/L)	3.6±0.7	4.1±0.8	4.3±0.5	＜0.001
TG(mmol/L)	1.4±0.4	1.6±0.3	1.5±0.4	0.038
HDL(mmol/L)	1.0±0.2	1.1±0.3	1.0±0.2	0.362
LDL(mmol/L)	2.5±0.8	2.6±0.6	2.5±0.4	0.553
ALT(U/L)	25.5±8.4	26.5±4.8	26.3±5.9	0.648
AST(U/L)	23.2±5.6	24.8±3.1	24.5±4.6	0.156
Scr(μmol/L)	58.0±8.1	59.8±6.1	59.6±6.0	0.27
FT4I(pmol/L)	14.4±1.6	15.0±2.5	14.9±2.2	0.212

HbA1c (%)	5.0±0.4	5.9±0.3	7.5±0.6	＜0.001
HbA1c(mmol/mol)	31.6±4.5	40.6±2.9	57.8±5.9	＜0.001
GLU(mmol/L)	4.8±0.5	6.2±0.3	8.2±1.0	＜0.001
OGTT 2h(mmol/L)	6.6±0.4	8.6±0.9	14.4±1.8	＜0.001

BMI: body mass index; TC: total cholesterol; TG: triglyceride; HDL: high density lipoprotein; LDL: low density lipoprotein; ALT: alanine transaminase; AST: aspartate transaminase; Scr: serum creatinine, FT4I: free thyroxine index; GLU: glucose; OGTT: oral glucose tolerance test
