# Supplementary material for: Hsa_circ_0054633 in peripheral blood can be used as a diagnostic biomarker of pre-diabetes and type 2 diabetes mellitus
Source: Acta Diabetol. 2016 Nov 23;54(3):237–45. doi: 10.1007/s00592-016-0943-0 (PMC5329094; doi:10.1007/s00592-016-0943-0)
Supplement: Supplementary file 2 — Supplementary material 2 (PDF 7 kb) [file 592_2016_943_MOESM2_ESM.pdf]

Supplementary Table 4. Nucleotide sequences of primers used for Q-RCR

	Forward	Reverse	Product length
hsa_circ_0068087	TCATTCCTCTATTTGTACAGTGGCT	GGCCCCTCAGTGTACGTCTT	141
hsa_circ_0054633	TTGCTTTCTACACTTTCAGGTGAC	GCTTTTTGTCTGTAGTCAACCACCA	110
hsa_circ_0124636	TTGCATTGTGGGCGGTATGC	TCCCCGGGTATACAAAAGTGAGA	127
hsa_circ_0139110	CCAAGCAGTCACAGAAGCTGG	ATACAGGCACCCAGGTAGGC	148
hsa_circ_0018508	TCTTTGCCACATATTGGGTGACT	ACACCAGGTACCGGTTATCCA	111
hGAPDH	TGTTGCCATCAATGACCCCTT	CTCCACGACGTACTCAGCG	202
