# Supplementary material for: Hsa_circ_0054633 in peripheral blood can be used as a diagnostic biomarker of pre-diabetes and type 2 diabetes mellitus
Source: Acta Diabetol. 2016 Nov 23;54(3):237–45. doi: 10.1007/s00592-016-0943-0 (PMC5329094; doi:10.1007/s00592-016-0943-0)
Supplement: Supplementary file 3 — Supplementary material 3 (PDF 127 kb) [file 592_2016_943_MOESM3_ESM.pdf]

Supplementary Table 5. Differential expressed circRNAs in the T2DM group of the first cohort.

Upregulated	*P* value	Fold change	Downregulated	*P* value	Fold change	Downregulated	*P* value
hsa_circ_0068087	0.008	3.2	hsa_circ_0024204	<0.001	2.1	hsa_circ_0129397	0.002
hsa_circ_0054633	0.005	2.9	hsa_circ_0038475	0.017	2.0	hsa-circRNA12505-55	0.041
hsa_circ_0124636	0.002	2.8	hsa_circ_0115355	<0.001	2.5	hsa_circ_0009070	0.004
hsa_circ_0061113	0.046	2.6	hsa_circ_0123377	<0.001	2.4	hsa_circ_0092672	0.003
hsa-circRNA13247-1	0.028	2.6	hsa_circ_0057526	<0.001	2.0	hsa_circ_0035593	0.005
hsa_circ_0118599	0.046	2.6	hsa-circRNA14362-13	0.007	2.0	hsa_circ_0054734	0.006
hsa-circRNA9652-1	0.021	2.6	hsa-circRNA12505-40	0.003	2.1	hsa_circ_0021780	<0.001
hsa_circ_0139110	0.009	2.6	hsa_circ_0099580	<0.001	3.0	hsa_circ_0071855	<0.001
hsa_circ_0062596	0.018	2.5	hsa_circ_0132149	<0.001	2.2	hsa-circRNA2177-30	<0.001
hsa_circ_0062195	0.046	2.5	hsa_circ_0099712	<0.001	2.1	hsa_circ_0016278	0.027
hsa_circ_0105688	0.022	2.5	hsa_circ_0002949	0.016	2.0	hsa_circ_0109315	<0.001
hsa_circ_0080192	0.040	2.5	hsa_circ_0119824	0.004	2.3	hsa_circ_0028010	0.004
hsa_circ_0018508	0.007	2.4	hsa-circRNA10051-8	0.007	2.3	hsa_circ_0140073	<0.001
hsa_circ_0133936	0.016	2.4	hsa_circ_0083544	0.013	2.1	hsa_circ_0039353	0.004
hsa_circ_0092267	0.038	2.4	hsa_circ_0120951	<0.001	2.6	hsa_circ_0029681	<0.001
hsa_circ_0081980	0.013	2.4	hsa_circ_0085163	<0.001	2.1	hsa-circRNA10165-8	<0.001
hsa_circ_0084597	0.012	2.4	hsa_circ_0139952	0.009	2.4	hsa-circRNA15265-3	<0.001
hsa_circ_0103926	0.011	2.4	hsa_circ_0138400	<0.001	2.1	hsa_circ_0094900	<0.001
hsa_circ_0066551	0.003	2.4	hsa-circRNA2177-25	<0.001	2.6	hsa-circRNA14910-11	<0.001
hsa_circ_0011082	0.048	2.3	hsa_circ_0087243	0.003	2.1	hsa_circ_0095180	0.048
hsa_circ_0082410	0.032	2.3	hsa_circ_0023761	<0.001	2.1	hsa_circ_0031862	0.011
hsa_circ_0119529	0.010	2.3	hsa_circ_0071857	<0.001	2.2	hsa-circRNA1958-5	0.012
hsa_circ_0003071	0.049	2.3	hsa-circRNA1958-2	0.002	2.7	hsa-circRNA5762-14	<0.001
hsa_circ_0069264	0.030	2.3	hsa_circ_0116853	0.004	2.1	hsa_circ_0071865	<0.001
hsa_circ_0111612	0.047	2.3	hsa_circ_0085080	0.002	2.1	hsa-circRNA6396-26	0.008
hsa_circ_0075700	0.028	2.3	hsa_circ_0126845	<0.001	2.1	hsa_circ_0087215	<0.001
hsa_circ_0077770	0.003	2.3	hsa_circ_0085445	0.017	2.1	hsa-circRNA10329-63	0.041
hsa_circ_0120470	0.044	2.3	hsa_circ_0005684	<0.001	2.1	hsa_circ_0117148	<0.001
hsa_circ_0006010	0.009	2.2	hsa_circ_0000673	<0.001	2.6	hsa_circ_0120341	0.020
hsa_circ_0069862	0.019	2.2	hsa_circ_0094904	<0.001	2.6	hsa_circ_0053327	0.003
hsa-circRNA4746-8	0.030	2.2	hsa_circ_0134682	0.017	2.0	hsa_circ_0027801	0.001
hsa_circ_0054129	0.001	2.2	hsa_circ_0021570	0.002	2.1	hsa_circ_0025977	<0.001
hsa_circ_0086623	0.021	2.2	hsa_circ_0074770	<0.001	2.1	hsa-circRNA15591-21	0.008
hsa_circ_0007858	0.049	2.2	hsa_circ_0058526	<0.001	2.1	hsa-circRNA3081-40	<0.001
hsa_circ_0121546	0.022	2.2	hsa_circ_0030737	<0.001	2.1	hsa_circ_0004083	0.014
hsa_circ_0026158	0.016	2.2	hsa_circ_0028009	0.002	2.8	hsa-circRNA5762-18	0.001
hsa_circ_0028886	0.026	2.2	hsa_circ_0103024	<0.001	2.2	hsa_circ_0007090	0.008
hsa_circ_0086409	0.009	2.2	hsa-circRNA12910-7	<0.001	2.1	hsa_circ_0025973	<0.001
hsa_circ_0054647	0.033	2.2	hsa_circ_0071841	<0.001	2.1	hsa_circ_0060232	0.042
hsa-circRNA11217	0.017	2.2	hsa_circ_0123376	<0.001	2.1	hsa_circ_0119707	0.003
hsa_circ_0030004	0.008	2.2	hsa_circ_0120335	0.009	2.1	hsa-circRNA7969-7	0.006
hsa_circ_0133005	0.001	2.2	hsa_circ_0012364	0.017	2.1	hsa_circ_0131079	<0.001
hsa-circRNA15423-15	0.029	2.2	hsa_circ_0086460	0.006	2.2	hsa_circ_0001347	0.004
hsa_circ_0043382	0.032	2.1	hsa_circ_0028967	0.001	2.1	hsa_circ_0136924	0.031
hsa_circ_0080517	0.015	2.1	hsa-circRNA6282-41	0.012	2.0	hsa_circ_0044870	<0.001
hsa_circ_0128150	0.012	2.1	hsa_circ_0067587	<0.001	2.1	hsa_circ_0047632	0.028
hsa_circ_0057701	0.045	2.1	hsa-circRNA949-20	<0.001	2.0	hsa_circ_0101921	0.014
hsa_circ_0033065	0.034	2.1	hsa_circ_0053664	<0.001	2.1	hsa_circ_0024206	0.007
hsa_circ_0060806	0.038	2.1	hsa_circ_0135645	0.003	3.0	hsa-circRNA15511-9	0.015
hsa_circ_0102413	0.009	2.1	hsa_circ_0054554	0.021	2.1	hsa_circ_0071390	0.003
hsa_circ_0028442	0.026	2.1	hsa_circ_0117150	<0.001	2.0	hsa_circ_0038470	<0.001
hsa_circ_0021637	0.037	2.1	hsa_circ_0115373	<0.001	2.2	hsa_circ_0134173	0.004
hsa_circ_0122759	0.002	2.1	hsa_circ_0037041	<0.001	2.1	hsa_circ_0099702	<0.001
hsa_circ_0126861	0.001	2.1	hsa-circRNA3310-95	0.005	2.0	hsa_circ_0119710	0.002

hsa_circ_0047479	0.007	2.1	hsa-circRNA12505-5	0.003	2.1	hsa_circ_0129029	0.007
hsa_circ_0129230	0.026	2.1	hsa_circ_0001467	<0.001	2.4	hsa_circ_0094899	0.003
hsa_circ_0133090	0.017	2.1	hsa_circ_0064736	<0.001	2.0	hsa_circ_0023779	<0.001
hsa_circ_0136091	0.020	2.1	hsa_circ_0066868	<0.001	2.0	hsa-circRNA15564-3	0.003
hsa_circ_0010879	0.008	2.1	hsa_circ_0013616	<0.001	2.1	hsa_circ_0132592	0.011
hsa_circ_0100191	0.017	2.1	hsa_circ_0028966	<0.001	2.1	hsa_circ_0040358	0.019
hsa_circ_0050044	0.040	2.1	hsa_circ_0092511	0.013	2.4	hsa_circ_0105527	<0.001
hsa_circ_0054646	0.012	2.1	hsa_circ_0053636	0.027	2.0	hsa_circ_0066232	0.003
hsa_circ_0090453	0.039	2.0	hsa_circ_0025979	<0.001	2.1	hsa_circ_0117152	<0.001
hsa_circ_0061012	0.009	2.0	hsa-circRNA6213-4	<0.001	2.1	hsa_circ_0035597	0.009
hsa_circ_0130694	0.034	2.0	hsa_circ_0085446	0.003	2.0	hsa_circ_0121926	0.035
hsa-circRNA3289	0.007	2.0	hsa_circ_0119159	<0.001	2.1	hsa_circ_0054555	<0.001
hsa_circ_0048884	0.003	2.0	hsa_circ_0067586	<0.001	2.1	hsa_circ_0025985	<0.001
hsa_circ_0126764	0.017	2.0	hsa_circ_0031047	0.012	2.2	hsa_circ_0041697	<0.001
hsa-circRNA12986-3	0.044	2.0	hsa_circ_0089925	0.005	2.3	hsa_circ_0000544	<0.001
hsa_circ_0106442	0.032	2.0	hsa_circ_0108704	0.019	2.1	hsa_circ_0110429	<0.001
hsa_circ_0026455	0.027	2.0	hsa_circ_0054812	0.001	2.2	hsa_circ_0049355	0.014
hsa-circRNA5933-3	0.017	2.0	hsa-circRNA12883-19	<0.001	2.1	hsa_circ_0076215	0.019
hsa_circ_0023866	0.031	2.0	hsa_circ_0056990	0.014	2.3	hsa_circ_0054731	<0.001
hsa_circ_0061787	0.026	2.0	hsa_circ_0015676	0.004	2.0	hsa_circ_0140074	0.003
hsa_circ_0092576	0.048	2.0	hsa_circ_0096843	0.003	2.4	hsa_circ_0117173	0.005
hsa_circ_0065692	0.024	2.0	hsa_circ_0057529	<0.001	2.1	hsa-circRNA8659-9	<0.001
hsa_circ_0111976	0.005	2.0	hsa_circ_0098946	<0.001	2.1	hsa_circ_0120602	0.004
hsa_circ_0128506	0.006	2.0	hsa_circ_0002316	0.026	2.0	hsa_circ_0119827	<0.001
			hsa-circRNA16072-9	<0.001	2.2	hsa_circ_0120560	<0.001
			hsa_circ_0047877	<0.001	2.0	hsa_circ_0017335	<0.001
			hsa_circ_0071866	<0.001	2.1	hsa_circ_0024215	<0.001
			hsa_circ_0140077	<0.001	2.6	hsa_circ_0115372	<0.001
			hsa_circ_0126894	0.003	2.2	hsa_circ_0093710	0.005
			hsa_circ_0134291	0.002	2.0	hsa_circ_0095347	0.003
			hsa-circRNA12505-2	<0.001	2.3	hsa_circ_0037889	<0.001
			hsa_circ_0091155	<0.001	2.2	hsa-circRNA7944-6	0.010
			hsa_circ_0129896	<0.001	2.3	hsa-circRNA10450-30	0.002
			hsa_circ_0101628	0.003	2.1	hsa_circ_0071229	<0.001
			hsa_circ_0040028	<0.001	2.5	hsa_circ_0127538	0.015
			hsa_circ_0118394	0.001	2.0	hsa-circRNA12505-31	<0.001
			hsa-circRNA12505-26	<0.001	2.2	hsa_circ_0047373	0.009
			hsa_circ_0030740	<0.001	3.1	hsa_circ_0129396	<0.001
			hsa_circ_0054778	<0.001	2.7	hsa_circ_0140563	<0.001
			hsa_circ_0003905	0.001	2.2	hsa_circ_0073470	<0.001
			hsa_circ_0069228	<0.001	2.4	hsa_circ_0038472	<0.001
			hsa-circRNA14020-21	<0.001	2.2	hsa-circRNA15591-31	0.002
			hsa_circ_0117499	<0.001	2.1	hsa_circ_0083837	0.034
			hsa_circ_0071853	<0.001	2.1	hsa_circ_0106175	0.004
			hsa_circ_0016817	0.027	2.2	hsa_circ_0124317	0.007
			hsa_circ_0069533	0.003	2.0	hsa_circ_0053760	0.006
			hsa_circ_0117183	<0.001	2.0	hsa_circ_0052756	0.003
			hsa_circ_0118288	0.008	2.1	hsa_circ_0105886	<0.001
			hsa_circ_0091154	<0.001	2.0	hsa_circ_0030731	<0.001

Fold change	Downregulated	*P* value	Fold change	Downregulated	*P* value	Fold change
2.2	hsa_circ_0009133	0.006	2.1	hsa_circ_0094884	<0.001	2.0
2.0	hsa_circ_0047651	<0.001	2.0	hsa_circ_0127463	<0.001	2.1
2.1	hsa_circ_0111501	<0.001	2.0	hsa_circ_0083169	<0.001	2.0
2.1	hsa_circ_0089932	0.019	2.1	hsa_circ_0096518	<0.001	2.1
2.0	hsa_circ_0088295	<0.001	2.0	hsa_circ_0023757	0.002	2.1
3.1	hsa_circ_0077656	<0.001	2.0	hsa-circRNA5011-23	<0.001	2.2
2.4	hsa_circ_0054095	<0.001	2.0	hsa_circ_0023780	<0.001	2.1
2.1	hsa_circ_0120954	0.001	2.7	hsa_circ_0084642	0.004	2.5
2.6	hsa_circ_0104186	0.003	2.5	hsa-circRNA10656-34	<0.001	2.1
2.3	hsa_circ_0140078	<0.001	2.2	hsa-circRNA12520-172	<0.001	2.1
2.0	hsa_circ_0114306	0.024	2.0	hsa_circ_0105778	0.001	2.2
2.4	hsa-circRNA14317-3	<0.001	2.3	hsa_circ_0098355	0.004	2.5
2.2	hsa_circ_0114302	<0.001	2.4	hsa-circRNA409-3	<0.001	2.2
2.3	hsa-circRNA10051-11	0.016	2.1	hsa_circ_0104551	<0.001	2.1
2.0	hsa_circ_0028968	0.002	2.2	hsa_circ_0030739	<0.001	2.6
2.2	hsa_circ_0009036	<0.001	2.2	hsa_circ_0120604	0.005	2.1
2.0	hsa_circ_0015678	0.002	2.1	hsa_circ_0106657	0.002	2.1
2.3	hsa_circ_0099436	0.031	2.6	hsa_circ_0057764	0.016	2.4
2.1	hsa_circ_0053329	<0.001	2.2	hsa_circ_0099711	<0.001	3.1
2.0	hsa_circ_0071220	<0.001	2.1	hsa_circ_0099990	<0.001	2.1
2.1	hsa_circ_0127531	0.045	2.8	hsa_circ_0033112	<0.001	2.1
2.0	hsa_circ_0028965	<0.001	2.1	hsa-circRNA11806-28	<0.001	2.7
2.0	hsa_circ_0028008	0.008	2.2	hsa_circ_0132239	0.002	2.1
2.3	hsa_circ_0023773	0.001	2.2	hsa_circ_0104275	<0.001	2.1
2.1	hsa_circ_0107665	0.022	2.4	hsa_circ_0054730	0.002	2.2
2.0	hsa_circ_0038250	0.001	2.3	hsa-circRNA2591-9	0.002	2.0
2.0	hsa_circ_0138403	<0.001	2.1	hsa-circRNA7550-33	<0.001	2.1
2.3	hsa_circ_0053641	<0.001	2.3	hsa-circRNA2198-7	0.007	2.4
2.3	hsa_circ_0139201	<0.001	2.0	hsa_circ_0120323	<0.001	2.0
2.2	hsa_circ_0037887	<0.001	2.5	hsa_circ_0120339	<0.001	2.2
2.2	hsa_circ_0031939	0.044	2.3	hsa_circ_0008855	<0.001	2.2
2.2	hsa-circRNA6756-36	<0.001	2.0	hsa-circRNA4610-36	<0.001	2.1
2.3	hsa_circ_0069397	<0.001	2.1	hsa_circ_0120342	0.001	2.1
2.0	hsa_circ_0140287	<0.001	2.1	hsa_circ_0054761	<0.001	2.3
2.0	hsa_circ_0067588	<0.001	2.1	hsa-circRNA12505-51	0.002	2.2
2.3	hsa-circRNA7697-20	0.019	2.3	hsa-circRNA12520-126	<0.001	2.3
2.1	hsa_circ_0130488	<0.001	2.1	hsa_circ_0041155	0.003	2.0
2.4	hsa-circRNA2198-13	0.003	2.7	hsa_circ_0084643	0.007	2.4
2.0	hsa_circ_0054728	0.004	2.1	hsa_circ_0120607	<0.001	2.3
2.1	hsa_circ_0138402	0.001	2.0	hsa_circ_0027702	0.018	2.6
2.1	hsa-circRNA16072-18	<0.001	2.0	hsa_circ_0075564	<0.001	2.3
2.0	hsa_circ_0069532	0.016	2.1	hsa-circRNA8505-7	<0.001	2.2
2.2	hsa-circRNA12693-13	<0.001	2.0	hsa_circ_0140076	<0.001	2.4
2.1	hsa_circ_0026573	0.010	2.4	hsa_circ_0138382	<0.001	2.4
2.1	hsa-circRNA9015-34	<0.001	2.6	hsa_circ_0071858	<0.001	2.1
2.2	hsa-circRNA3487-6	<0.001	2.4	hsa_circ_0029812	0.017	2.1
2.3	hsa_circ_0075045	0.003	2.0	hsa_circ_0031081	0.030	2.0
2.2	hsa-circRNA10767	0.006	2.0	hsa_circ_0030732	<0.001	2.6
2.0	hsa_circ_0072732	0.002	2.4	hsa_circ_0015672	0.005	2.0
2.1	hsa_circ_0127462	<0.001	2.0	hsa_circ_0111502	<0.001	2.2
2.2	hsa_circ_0105010	<0.001	2.5	hsa_circ_0134171	0.005	2.3
2.1	hsa_circ_0081889	<0.001	2.1	hsa_circ_0016397	<0.001	2.4
2.1	hsa_circ_0129104	0.022	2.6	hsa_circ_0001168	0.005	2.1
2.3	hsa-circRNA15514-9	<0.001	2.1	hsa-circRNA10072-4	<0.001	2.0

2.0	hsa_circ_0085406	<0.001	2.7	hsa-circRNA7550-9	<0.001	2.1
2.9	hsa_circ_0084645	0.006	2.5	hsa-circRNA5854-5	0.010	2.0
2.2	hsa_circ_0133355	0.007	2.5	hsa_circ_0022301	0.003	2.2
2.0	hsa-circRNA12520-159	0.001	2.2	hsa-circRNA6489-15	<0.001	2.1
2.1	hsa-circRNA4380-15	<0.001	2.1	hsa_circ_0120605	0.008	2.0
2.0	hsa_circ_0136904	<0.001	2.4	hsa-circRNA3081-2	<0.001	2.1
3.2	hsa_circ_0039423	0.001	2.1	hsa-circRNA7104-135	<0.001	2.0
2.2	hsa_circ_0069410	0.014	2.0	hsa_circ_0122354	0.002	2.0
2.0	hsa_circ_0013489	<0.001	2.3	hsa-circRNA4388-10	0.003	2.3
2.0	hsa_circ_0005567	0.007	2.0	hsa_circ_0087212	<0.001	2.3
2.1	hsa_circ_0136060	0.031	2.0	hsa_circ_0140534	<0.001	2.3
2.6	hsa-circRNA6510-1	<0.001	2.4	hsa_circ_0140292	<0.001	2.6
2.2	hsa_circ_0057280	0.016	2.4	hsa-circRNA7697-35	0.004	2.0
2.6	hsa_circ_0073828	<0.001	2.0	hsa_circ_0092459	0.007	2.0
2.0	hsa_circ_0126693	0.004	2.2	hsa_circ_0079321	0.024	2.0
2.1	hsa-circRNA9640-24	<0.001	2.0	hsa_circ_0077414	0.008	2.0
2.3	hsa_circ_0085444	0.002	2.1	hsa_circ_0112542	0.001	2.1
2.4	hsa-circRNA15511-5	0.004	2.2	hsa_circ_0025441	0.003	2.7
2.1	hsa-circRNA9274-3	0.023	2.0	hsa_circ_0047630	<0.001	2.6
2.0	hsa_circ_0001494	0.013	2.2	hsa_circ_0120559	<0.001	2.1
2.1	hsa_circ_0001774	0.001	2.0	hsa_circ_0022919	0.031	2.4
2.2	hsa_circ_0054985	<0.001	2.2	hsa_circ_0076400	<0.001	2.1
2.1	hsa_circ_0066238	<0.001	2.2	hsa_circ_0031863	0.010	2.0
2.2	hsa_circ_0127537	<0.001	2.2	hsa-circRNA12505-15	0.001	2.5
2.3	hsa_circ_0137811	<0.001	2.1	hsa_circ_0054726	<0.001	2.4
2.2	hsa_circ_0130333	<0.001	2.2	hsa_circ_0120606	<0.001	2.2
2.1	hsa_circ_0007018	<0.001	2.2	hsa_circ_0000849	<0.001	2.1
2.2	hsa_circ_0078516	0.004	2.1	hsa_circ_0135075	<0.001	2.1
2.4	hsa_circ_0119156	0.014	2.3	hsa_circ_0122182	0.002	2.1
2.3	hsa_circ_0055894	<0.001	2.0	hsa-circRNA7619-1	0.034	2.2
2.1	hsa-circRNA14588-10	<0.001	2.2	hsa_circ_0094898	<0.001	2.1
2.4	hsa_circ_0011826	<0.001	2.1	hsa_circ_0053665	0.002	2.1
2.3	hsa_circ_0060740	<0.001	2.2	hsa_circ_0126352	0.047	2.3
2.0	hsa_circ_0027365	<0.001	2.1	hsa_circ_0051623	0.048	2.0
2.2	hsa_circ_0075043	0.007	3.1	hsa_circ_0140081	<0.001	2.5
2.2	hsa_circ_0135599	<0.001	2.1	hsa_circ_0034370	0.004	2.0
2.1	hsa_circ_0029870	<0.001	2.1	hsa_circ_0072730	0.002	2.3
2.3	hsa_circ_0128169	0.019	2.2	hsa_circ_0071250	0.002	3.1
2.0	hsa-circRNA12520-31	0.002	2.2	hsa_circ_0105779	<0.001	2.3
2.6	hsa-circRNA9834-7	0.024	2.0	hsa_circ_0038424	0.002	2.1
2.0	hsa_circ_0117172	0.009	2.1	hsa_circ_0069885	0.003	2.0
2.3	hsa_circ_0074588	<0.001	2.1	hsa-circRNA11783-2	<0.001	2.4
2.4	hsa_circ_0034966	0.018	2.0	hsa-circRNA4380-6	<0.001	2.0
2.0	hsa_circ_0140080	<0.001	2.2	hsa_circ_0053657	<0.001	2.1
2.3	hsa-circRNA12520-47	<0.001	2.5	hsa-circRNA3069-7	<0.001	2.2
2.1	hsa_circ_0127442	<0.001	2.0	hsa_circ_0126839	<0.001	2.2
2.0	hsa_circ_0119266	<0.001	2.4	hsa-circRNA13479-39	0.005	2.8
2.4	hsa_circ_0072733	0.005	2.0	hsa_circ_0016790	0.004	2.9
2.5	hsa_circ_0062796	0.037	2.0			
